# Influence of Reactive Oxygen Species on Wound Healing and Tissue Regeneration in Periodontal and Peri-Implant Tissues in Diabetic Patients

**DOI:** 10.3390/antiox12091787

**Published:** 2023-09-21

**Authors:** Prima Buranasin, Hiromi Kominato, Koji Mizutani, Risako Mikami, Natsumi Saito, Kohei Takeda, Takanori Iwata

**Affiliations:** 1 Department of Conservative Dentistry and Prosthodontics, Faculty of Dentistry, Srinakharinwirot University, Bangkok 10110, Thailand; 2 Department of Periodontology, Graduate School of Medical and Dental Sciences, Tokyo Medical and Dental University (TMDU), 1-5-45 Yushima, Bunkyo-ku, Tokyo 113-8549, Japan

**Keywords:** diabetes, oxidative stress, periodontitis, reactive oxygen species, wound healing

## Abstract

Diabetes mellitus (DM) is associated with periodontal disease. Clinically, periodontal treatment is less effective for patients with DM. Oxidative stress is one of the mechanisms that link DM to periodontitis. The production of reactive oxygen species (ROS) is increased in the periodontal tissues of patients with DM and is involved in the development of insulin resistance in periodontal tissues. Insulin resistance decreases Akt activation and inhibits cell proliferation and angiogenesis. This results in the deterioration of wound healing and tissue repair in periodontal tissues. Antioxidants and insulin resistance ameliorants may inhibit ROS production and improve wound healing, which is worsened by DM. This manuscript provides a comprehensive review of the most recent basic and clinical evidence regarding the generation of ROS in periodontal tissues resulting from microbial challenge and DM. This study also delves into the impact of oxidative stress on wound healing in the context of periodontal and dental implant therapies. Furthermore, it discusses the potential benefits of administering antioxidants and anti-insulin resistance medications, which have been shown to counteract ROS production and inflammation. This approach may potentially enhance wound healing, especially in cases exacerbated by hyperglycemic conditions.

## 1. Introduction: Oxidative Stress in Periodontitis

### 1.1. Basic and Clinical Findings

Reactive oxygen species (ROS) are free oxygen radicals, including superoxide anion radicals (O_2_^•−^), hydroxyl radicals (^•^OH), and other nonradical oxygen derivatives involved in oxygen radical production, such as hydrogen peroxide (H_2_O_2_), hyperchlorous acid (HOCl), and singlet oxygen (^1^O_2_) [1]. ROS are essential for normal cellular metabolism and biological processes and are constantly generated by tissue cells. However, high levels and prolonged presence of ROS, leading to oxidative stress, may worsen systemic inflammatory conditions and directly induce severe tissue damage involving lipid peroxidation, DNA and protein damage, and the oxidation of critical enzymes [2], leading to cell death. Typically, another class of substances within cells, known as antioxidants, can effectively interfere with or inhibit ROS-induced oxidative stress [2]. In normal physiological states, antioxidants efficiently counteract ROS, thus preventing ROS-induced tissue damage and deterioration. Over the past decade, oxidative stress has been reported to be involved in the etiology, pathogenesis, and progression of many chronic inflammatory diseases, including periodontitis [2].

Periodontal disease is a multifactorial inflammatory condition that results in the loss of tooth-supporting tissue. In addition to the immune-inflammatory responses involved in the onset and progression of periodontitis, oxidative stress has been garnering growing attention. Previous in vitro studies have demonstrated an increase in oxidative stress in human gingival fibroblasts (HGFs) [3] and human periodontal ligament (PDL) fibroblasts [4] after exposure to lipopolysaccharide (LPS) from *Porphyromonas gingivalis.* Various examinations have revealed increased levels of oxidative stress markers in the saliva, gingival crevicular fluid, and plasma of patients with periodontitis, supporting the relationship between oxidative stress and periodontal inflammation.

### 1.2. Diabetes Mellitus (DM)-Induced ROS Production in Periodontal Tissues

DM, a metabolic disorder characterized by hyperglycemia, is a risk factor for delayed wound healing in periodontitis [5]. The association between these two diseases was verified as a two-way relationship [6]. Increased oxidative stress contributes substantially to the etiopathogenesis of DM and periodontal disease [7]. Studies have suggested that insulin resistance, induced by oxidative stress in the gingiva of rodent models with pre-diabetes, leads to vascular inflammation and periodontal destruction [8]. Moreover, increased levels of advanced glycation end products (AGEs) have been reported in the gingival tissue of patients with DM compared to that in non-DM individuals, enhancing oxidative stress and escalating tissue injury [9,10]. 

**Figure 1:**
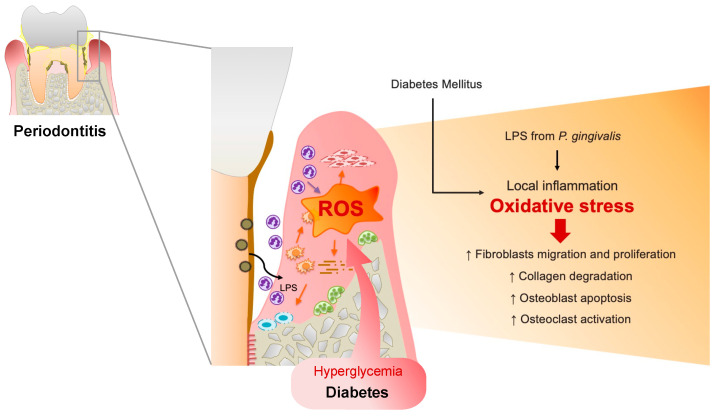
Upregulated reactive oxygen species (ROS) in the periodontal tissues of patients with DM. In a microbial invasion, fragments of periodontal pathogens such as *P. gingivalis* and lipopolysaccharides (LPS) invade gingival tissues, activating the inflammatory response. Polymorphonuclear neutrophils (PMNs) gather in the gingiva sulcus and periodontal tissues, release inflammatory mediators, and cause the overproduction of ROS, resulting in oxidative stress within the tissues. ROS can trigger cytotoxic effects on periodontal-resident cells through fibroblast migration and proliferation, induce osteoblast apoptosis, and activate osteoclasts. Furthermore, ROS can damage proteoglycan, hyaluronan, and collagen, leading to connective tissue breakdown and impairing wound healing in periodontal disease. Similarly, in DM conditions, hyperglycemia-induced oxidative stress in periodontal tissue results in delayed gingival wound healing in patients with periodontitis and DM.

Furthermore, systemic oxidative stress levels were elevated in periodontitis patients [11], and periodontitis is associated with increased oxidative stress and compromised glycemic control in patients with Type 2 DM [12], which may explain the increased vulnerability of patients with DM to periodontitis and the progression to advanced periodontitis in patients with DM than in patients without DM (Figure 1).

## 2. Basic Research on the Influence of Oxidative Stress on Periodontitis

### 2.1. ROS Upregulation Impairs Wound Healing in the Periodontal Tissues

ROS play crucial roles in antimicrobial defense mechanisms, cell signaling, and gene regulation [13,14]. ROS overproduction leads to an increased oxidant load and diminished antioxidant capacity, resulting in oxidative stress within tissues [15]. Additionally, ROS generation is promoted during constant bacterial challenge in the periodontal tissues. Polymorphonuclear neutrophils (PMNs) are critical components of the innate immune system as the first inflammatory cells to gather in the gingival sulcus and periodontal tissues [16]. In addition to protecting the periodontium from microbial invasion, these cells release potent lysosomal enzymes and ROS that potentially trigger cytotoxic effects and facilitate hard tissue deterioration through osteoclastogenesis, which is responsible for the progression of periodontal disease [17,18]. Moreover, ROS are the end products of the mitochondrial respiratory burst in PMN during phagocytosis [2] and are considered the predominant source of ROS in periodontitis [19,20]. The successful healing of periodontal wounds relies on a coordinated process that engages various cell types within the periodontium. These include keratinocytes, fibroblasts, endothelial cells, macrophages, and platelets. Notably, fibroblasts, which are the most prevalent cells in connective tissue, play a pivotal role in orchestrating the wound healing process within the periodontium. ROS mainly act through lipid peroxidation [14,21] and damage proteins [22] and DNA [14,23]. Therefore, the interactions between ROS and lipid peroxidation products significantly influence fibroblast adhesion, proliferation, and viability. In normal conditions, collagen and other extracellular matrix components play a crucial role in regulating the function of connective tissue cells. On the other hand, the generation of ROS can damage proteoglycans, hyaluronan, and collagen, leading to the breakdown of connective tissues [2] and impairing wound healing in periodontal tissue.

DM is a risk factor for periodontitis. Hyperglycemia, a state of high glucose concentration, is a significant factor responsible for the activation of oxidative stress [15,24]. Similarly, literature has reported impaired gingival wound healing in patients with periodontitis and DM. Various concentrations of glucose have been proposed to create an environment that mimics DM conditions in periodontal cells [25,26,27,28]. In a previous in vitro study, it was shown that hyperglycemia-induced oxidative stress inhibits cell proliferation and migration while leading to cell toxicity in HGFs. This effect was observed in conjunction with a significant increase in intracellular ROS levels in HGFs when they were exposed to media containing high glucose concentrations (50 and 75 mM). Furthermore, the impact of oxidative stress and cell toxicity was found to increase in a dose-dependent manner compared to those in normal conditioned media. Interestingly, elevated mRNA expression levels of oxidative stress markers, including nuclear factor erythroid 2-related factor 2 (Nrf2), heme oxygenase 1 (HO1), superoxide dismutase 1 (SOD1), and catalase (CAT), were also shown under high-glucose conditions [27].

Consistently, a significant decrease in cell migration and proliferation and an increase in the accumulation of ROS were observed in gingival fibroblasts from rats with DM. In the same study, the gene expression of Nox1, Nox2, Nox4, and p47 was upregulated in rats with DM compared to that in control rats in both in vitro and in vivo experiments, resulting in prolonged wound closure. In agreement with these studies, low epithelial wound coverage, reduced new connective tissue formation, and decreased fibroblast density were observed in the gingival wounds of mice with DM [29].

### 2.2. ROS Upregulation Inhibits the Osseointegration of Dental Implants

Recently, dental implants have replaced edentulous dentition. ROS overproduction induces dysfunction and apoptosis of osteoblasts [30] and activates osteoclasts [31], leading to defective bone regeneration at the titanium-bone interface. Previous in vitro studies have reported that oxidative stress substantially impairs the proliferation, differentiation, and mineralization of osteoblasts. 

As previously mentioned, DM regulates ROS overproduction during periodontitis. Evidence also indicates compromised osteointegration owing to oxidative stress, which increases the risk of dental implant failure. In vitro studies have shown that DM exacerbates periodontal bone loss by enhancing osteoblast apoptosis and osteoclast formation in rats with ligature-induced periodontitis [32]. Mesenchymal stem cells are stimulated to differentiate into osteoblasts within 1 to 6 days after implantation [33]. A recent study on rat bone marrow-derived mesenchymal stem cells (BMMSCs) under hyperglycemic conditions revealed ROS overexpression, leading to decreased proliferative capability and inhibition of the calcification process on titanium discs [34].

Due to the slower recovery of alveolar bone defects compared to that of bones [35], experimental models were needed to validate the impacts of DM on bone healing in the alveolar bone. Several studies reported oral implant osseointegration and bone regeneration in an experimental alveolar ridge tooth extraction model [36,37,38]. A prior study discovered that there was a significant generation of ROS both in diabetic serum and within osteoblasts located on porous titanium implants (pTi). This ROS production had the potential to result in substantial osteoblast dysfunction, ultimately impairing the process of bone ingrowth into pTi in DM rabbits [39]. Interestingly, a recent study showed compromised osseointegration of titanium implants in the maxilla of rats with diabetes, with increased oxidative stress markers, such as p47 and Nox2, and decreased SOD1 in the bone adjacent to the implant in the DM group [34].

### 2.3. Antioxidants Recover Wound Healing In Vitro and In Vivo Studies

Oxidative stress occurs only when ROS and antioxidants are imbalanced, and antioxidants cannot neutralize ROS overproduction [40]. Reduced SOD1 and CAT activities and cell apoptosis have been observed in human gingival tissue with increasing periodontal pocket depth [41]. Antioxidants play a critical role in the defense against ROS. Accordingly, studies have examined the application of antioxidants in the treatment of periodontitis. However, only a few studies have investigated the protective role of antioxidants in the wound healing of periodontal-resident cells. 

N-acetylcysteine (NAC), a widely employed antioxidant, has garnered significant attention as a therapeutic agent owing to its robust antioxidant properties [42]. The pharmacological and biological attributes of NAC, encompassing anti-inflammatory, antimicrobial, and antioxidant activities, position it as a promising therapeutic candidate for applications within the dental field [43]. In vitro studies suggested that applying NAC to the culture could restore osteoblast dysfunction to a near-normal level because of the improved redox balance [44]. Resveratrol suggests antioxidant effects by nearly abrogating ROS production, enhancing cell proliferation, promoting type I collagen synthesis, and facilitating mitochondrial respiration in HGFs better than quercetin and NAC [45]. 

NAC prevents LPS-induced ROS production in HGFs [46]. Furthermore, NAC treatment notably mitigated the intracellular ROS generation enhanced by cyclic mechanical stress in human periodontal ligament stem cells (PDLSCs). This was achieved through the downregulation of Nrf2 expression, ultimately leading to improved osteogenic differentiation [42]. Furthermore, in DM conditions, NAC treatment restores cell migration, improves proliferation, and partially reverses cellular damage in high glucose-induced oxidative stress in HGFs [27]. Resveratrol has showcased remarkable antioxidant effects in comparison to quercetin and NAC. It has proven effective in reducing ROS production, boosting cell proliferation, promoting collagen synthesis, and enhancing mitochondrial respiration in HGFs [45]. 

Metformin is the most commonly used antihyperglycemic drug in patients with type 2 DM. However, reports regarding the effects of metformin on periodontal wound healing are limited. A recent study demonstrated that metformin upregulated the expression of vascular endothelial growth factor (VEGF), which is related to angiogenesis and tissue formation through Akt activation, thereby improving cell migration and proliferation. Moreover, metformin administration improved the mRNA expression of eNOS, VEGF, and fibroblast growth factor 2 (FGF2) through insulin signaling in vivo. Thus, re-epithelialization and wound closure are significantly accelerated in metformin-treated pre-DM mice [47].

Enamel matrix derivatives (EMD) are widely used in periodontal regeneration [48,49]. EMD has been in clinical use for nearly three decades, and its capacity to regenerate periodontal tissues, encompassing cementum, periodontal ligament, and alveolar bone, has been well established [49]. A recent clinical study has further demonstrated that periodontal regenerative therapy utilizing EMD effectively enhances clinical outcomes, even in elderly patients with delayed healing [48]. EMD is extracted from developing porcine teeth and comprises over 90% of the protein complex, which is comprised of amelogenin and other peptides. These components play pivotal roles in enamel crystal formation during crown maturation and are involved in mediating acellular cementum formation as well as the attachment of the periodontal apparatus during root development [50]. Multiple in vitro studies have provided evidence that cells exposed to EMD establish a link between these effects and the potential benefits it offers in terms of periodontal wound healing and regeneration. In vitro studies have demonstrated the anti-inflammatory effects of EMD in human PDL cells [51] and HGFs [52]. In addition, EMD induces periodontal regeneration, as evidenced by the enhanced proliferation and cell-matrix interaction of human PDL cells [53], promotes the differentiation of osteogenic progenitor cells, and increases matrix production in rat gingival fibroblasts [54]. Following the application of EMD, several key mechanisms have been observed. There is a decreased production of interleukin-1b and interleukin-18, alongside increased levels of PGE2, with minimal changes in TNF-alpha expression. EMD also brings about a significant adjustment in the osteoprotegerin and RANKL balance, characterized by elevated osteoprotegerin levels and reduced RANKL levels, leading to reduced osteoclast formation and activity. Furthermore, EMD enhances the proliferation and migration of T lymphocytes, which facilitates tissue debridement by macrophages. Moreover, EMD promotes mesenchymal cell differentiation into hard tissue-forming cells and enhances periodontal ligament cell regeneration. It also improves the differentiation of microvascular cells and angiogenesis following its application. Additionally, studies have demonstrated that EMD lowers bacterial numbers, resulting in a diminished inflammatory state. Furthermore, EMD accelerates vascular endothelial growth factor (VEGF) expression in diabetic rats, thereby promoting periodontal tissue regeneration [55]. Additionally, a recent study on the EMD treatment of rat BMMSCs revealed a significant reduction in ROS production in high-glucose cultures, verifying the antioxidant effect of EMD [56]. In patients with periodontitis, there is an elevation in the levels of oxidative stress markers within gingival tissues. Additionally, individuals with diabetes are known to experience heightened systemic oxidative stress. Given that patients with diabetes are more susceptible to periodontitis and its exacerbations, oxidative stress is believed to potentially play a role in the underlying mechanisms. BMMSCs hold the potential for early-phase hard tissue formation. Previous studies have indicated a relationship between oxidative stress and BMMSCs, where the production of ROS has detrimental effects on the functionality of BMMSCs [57]. ROS production has also been linked to the increased expression of proinflammatory cytokines, which contribute to connective tissue destruction and bone resorption [2]. Furthermore, there is evidence to suggest that downregulating ROS is responsible for enhancing osteogenic differentiation in BMMSCs [58]. However, conditions associated with HG levels inhibit the function of BMMSCs and alkaline phosphatase activity [59]. Notably, EMD treatment contributes to BMMSC calcification by downregulating ROS, thereby affecting periodontal tissue regeneration, even in the presence of HG conditions. Particularly, the in vivo results showed reduced inflammatory cells and increased mRNA expression of SOD-1 in EMD-treated sites, even in rats with DM. Enhanced connective tissue attachment formation, cementum formation, and primary wound closure have been documented in EMD-treated sites, regardless of the control or rats with DM [56], contributing to the early phase of wound healing and periodontal tissue regeneration. This study suggests that the antioxidative effects of EMD contribute to the promotion of periodontal tissue regeneration by inhibiting high glucose-induced oxidative stress. To date, surgical periodontal therapy, including periodontal regenerative therapy, has not been widely recommended for patients with diabetes, primarily due to concerns about potential delays in wound healing. However, recent literature reports [60] have shown that regenerative therapy using EMD and minimally invasive techniques can achieve clinical outcomes in diabetic patients that are comparable to those in individuals without systemic health issues. This improved outcome may be attributed in part to the antioxidant effects of EMD.

## 3. Clinical Study 

### 3.1. Increased ROS in Patients with Periodontitis

Periodontal disease is caused by plaque biofilms and is an inflammatory process that results in the loss of periodontal tissue attachment to the root surfaces and adjacent alveolar bone, causing tooth loss. Previous clinical studies have reported elevated ROS levels in the periodontal tissues of patients with periodontitis. A clinical study conducted in Tunisia investigated biochemical parameters and oxidative stress markers in the plasma of healthy subjects and patients with chronic periodontitis. The results indicated a significant reduction in plasma antioxidant activities, including catalase, glutathione reductase (GR), and total antioxidant capacity (TAOC), among patients with periodontitis. This decrease in plasma antioxidant activity may contribute to the development of periodontal diseases [61].

Studies have established a link between periodontitis and oxidative stress markers in the saliva, gingival crevicular fluid, and plasma of patients with periodontal disease. Elevated levels of the lipid peroxidation marker malondialdehyde (MDA) have been reported in the serum, saliva, and gingival crevicular fluid of patients with periodontitis [14]. Moreover, the levels of 8-Hydroxy-deoxyguanosine (8OHdG) [11,62], MDA, nitric oxide (NO), and total oxidant status (TOS) [11] were elevated in the saliva of patients with periodontitis, and significantly higher levels of total protein carbonyls in patients with periodontitis were correlated with advanced periodontal attachment loss [22,63].

Previous studies have hypothesized that periodontitis induces oxidative stress and reduces antioxidant activity in periodontal tissues. Thiobarbituric acid reactive substances (TBARS) are enzymatic antioxidants acting as a series of antioxidant defense mechanisms to prevent the harmful effects of ROS. Patients with periodontitis had significantly higher TBARS levels than healthy subjects. In addition, the plasma, erythrocytes, erythrocyte membranes, and periodontal tissues of patients with periodontitis have been shown to have significantly higher levels of enzymatic antioxidant activity and lower levels of non-enzymatic antioxidants than those of healthy subjects [64]. In a recent clinical study, it was observed that the expression of genes responsible for encoding the antioxidant enzyme products, specifically glutathione peroxidase 1 (GPX1) and thioredoxin 1 (TXN1), was significantly higher in the saliva of patients with periodontal disease. Conversely, the expression of genes encoding SOD1, GPX1, and TXN1 in the gingival tissue was significantly lower in patients with periodontitis compared to the control group [65].

### 3.2. Patients with Both Periodontitis and DM

Hyperglycemia, a characteristic of DM, increases ROS [66]. Numerous biochemical and basic pathogenic pathways associated with hyperglycemia, such as glucose autoxidation, polyol pathways, prostanoid synthesis, and protein glycation, increase ROS production in patients with DM [67,68,69]. Elevated ROS levels promote apoptosis by increasing cytochrome C and caspase-3 [70]. Excess ROS production in DM increases inflammation [71] and may lead to the destruction of periodontal tissues in patients with DM. 

Oxidative stress is strongly linked to the development of chronic periodontitis in individuals with diabetes [72]. Specifically, patients with type 2 diabetes and and diabetic individuals with periodontitis exhibit significantly lower plasma small molecule antioxidant capacity (pSMAC) and significantly higher levels of protein carbonyls, a marker of oxidative stress, compared to non-diabetic patients with periodontitis. In addition, high-sensitivity C-reactive protein (hsCRP) levels are significantly higher, suggesting that periodontitis is associated with increased oxidative stress and worse glycemic control in patients with type 2 diabetes [12]. When comparing oxidative stress markers between patients with type 2 diabetes and periodontitis and healthy individuals with periodontitis, it was found that salivary 8-OHdG concentrations were significantly higher in patients with periodontitis, regardless of their systemic status. Additionally, serum AGE concentrations were reported to be significantly higher in the diabetic group compared to healthy subjects with a systemic status [73].

Given these findings, it is essential to explore the effects of oxidative stress on “inflammatory diseases” associated with chronic periodontitis [74]. Future studies should aim to investigate the intricate relationship among reactive oxygen species, antioxidants, and oxidative stress concerning periodontal disease and diabetes.

### 3.3. Beneficial Effects of Antioxidants in Periodontal Therapy

In general, non-surgical treatments such as scaling, root planing (SRP) [75], and periodontal surgery [76] effectively improve periodontitis by reducing the probing pocket depth (PPD). Previous systematic reviews have shown that the combination of SRP and different treatment modalities does not provide any additional benefits to the primary outcomes of periodontal treatment [75,76].

Clinical studies of antioxidant application with non-surgical periodontal therapy were summarized in Table 1. Abou et al. investigated the plasma total antioxidant capacity (TAC) of patients with ChP to determine the adjuvant effect of vitamin C in non-surgical periodontal therapy. The results showed that TAC levels in patients with ChP significantly decreased, and non-surgical treatment with SRP increased plasma TAC levels in patients with ChP and significantly improved clinical parameters. However, compared with SRP alone, there was no additional effect on clinical indices and TAC concentrations [77]. Kunsongkeit et al. conducted a study to assess the effects of adjuvant periodontal therapy combined with vitamin C administration (500 mg/day) in patients with chronic periodontal disease and uncontrolled type 2 diabetes mellitus. The study showed significant improvements in all periodontal parameters (PI, BOP, GI, PPD, and CAL) from baseline in both groups. However, there were no significant differences between the groups, indicating that vitamin C administration did not provide additional benefits for promoting periodontal status [78]. The potential antioxidant effects of vitamin C as an adjunctive treatment for periodontal disease warrant further investigation [78]. Mathur et al. also compared SRP alone and SRP plus lycopene, zinc, and selenium as antioxidants and elucidated no significant difference in salivary uric acid levels between SRP alone and SRP plus antioxidants, whereas there was a significant increase in salivary uric acid levels over time in the antioxidant combination group. A significant increase in salivary uric acid levels was observed in the combined antioxidant group over time [79]. Taalab et al. conducted a clinical evaluation to assess the efficacy of tea tree oil (*Melaleuca alternifolia*) gel applied in the periodontal pocket as an adjunct to SRP for the treatment of moderate periodontitis. The study reported significant improvements in all clinical parameters (PPD, CAL, GI, and BOP) in both groups [80]. Raut et al. compared the efficacy of Coenzyme Q10 (CoQ10) and tea tree oil gel as adjuncts to SRP and showed that both antioxidant treatments were effective in reducing the clinical markers (Gingival Index, GI; PPD; and Clinical Attachment Level, CAL) of ChP [81]. Kaipa et al. reported significant improvements in GI, BOP, PPD, and CAL in patients with ChP following SRP adjunctive with Spirulina as an antioxidant [82]. 

Furthermore, a systematic review by Merle et al. examined whether CoQ10 combined with non-surgical periodontitis treatment improved periodontal parameters in patients with ChP. Approximately half of the studies reported significant group differences in the PPD. Further high-quality randomized controlled trials (RCTs) are required in the future [83]. A systematic review by Chatzopoulos et al. evaluated the effects of herbal products on periodontal disease in patients with periodontitis and examined the effects of using herbal products with anti-inflammatory and antioxidant properties in the oral cavity. Herbal products used as adjuncts to SRP demonstrated better clinical outcomes than placebo or no adjuncts. When used with SRP, these products were equivalent to or superior to those of chlorhexidine. These results suggest that herbal oral care products may be helpful for the treatment of patients with chronic oral diseases. These results suggest that herbal oral care products play a pivotal role in periodontal disease management [84].

Conversely, combination therapy may be effective in patients with type 2 DM [6], which is associated with periodontal disease progression [85] and impaired postoperative healing. Mizutani et al. conducted a systematic review and meta-analysis to examine whether the use of antioxidants in combination with periodontal therapy improved the periodontal parameters in patients with type 2 DM. These results suggest that the combination of melatonin, resveratrol, and omega-3 fatty acids with cranberry juice, propolis, and aloe vera gel in periodontal therapy significantly improves periodontal parameters in patients with type 2 DM. These results indicate that using antioxidants in combination with non-surgical periodontal therapy may significantly reduce periodontal pocket depth [86].

Because of the antioxidant properties of EMD, as mentioned above [56], EMD may also have potential benefits for periodontal tissue regeneration therapy in periodontal patients with type 2 DM. Mizutani et al. reported that minimally invasive surgery combined with EMD resulted in significant clinical attachment gain and bone filling at comparable levels in the type 2 DM and non-DM groups [87]. Although various factors may have contributed to these results, the anti-inflammatory and antioxidant effects of EMD may have influenced the periodontal tissues of DM patients, which may be associated with delayed healing after surgery.

### 3.4. Dental Implants and Antioxidant

Dental implants are widely used as prosthetics for partially or entirely edentulous dentition, and their demand is rapidly increasing. The incidence of implant mucositis and peri-implantitis is on the rise. Recent research has delved into the pathophysiology of wound healing and the mechanisms of regeneration mediated by reactive oxygen species in peri-implantitis. Patients with peri-implantitis exhibit elevated levels of oxidative stress-related genes, including NADPH oxidase and NOX4, along with reduced expression of osteogenesis-related genes such as RUNX, Osterix, and b-Catenin, when compared to healthy individuals [88]. This suggests that bone loss in inflammatory conditions such as peri-implantitis may result from the dysregulation of the differentiation process of MSCs into osteoblasts, which is closely associated with ROS production.

Recent research has explored the effectiveness of employing antioxidants alongside implant therapy. It has been observed that the application of gels containing nanovitamins C and E as adjuncts to mechanical debridement in the treatment of peri-implant mucositis significantly reduces PI, BOP, and PPD [89]. The administration of vitamin C, an antioxidant, to patients with ChP disease promotes soft tissue wound healing after implantation [90]. Given the expected continued increase in the use of implant therapy, further investigation into antioxidant combination therapies is warranted.

It is worth noting that DM has been associated with an increased risk of peri-implantitis [91]. Hyperglycemic conditions can accelerate bone loss related to peri-implantitis by altering the microbiome and intensifying the local inflammatory response [92]. However, there is limited clinical data available regarding the effectiveness and underlying mechanisms of peri-implantitis treatment in patients with DM. The use of antioxidants in patients with diabetes may be a potential avenue for improving osseointegration during implant therapy, and we await further clinical data in the future.

## 4. Conclusions

Previous studies indicate that oxidative stress may be an essential factor in the pathogenesis of DM and periodontal diseases. Furthermore, the coexistence of these diseases may increase the pathological effects of oxidative stress. As the role of oxidative stress changes as the disease progresses, antioxidant therapy, which affects oxidative damage and inflammatory processes, may have various impacts at each stage of the complex pathogenesis [86]. 

Oxidative stress may be an essential factor in the progression of DM and periodontal disease. Based on the literature review, the comorbidity of both diseases may exacerbate the pathological effects of oxidative stress in periodontal tissues. Additionally, antioxidants and anti-insulin resistance medications can inhibit ROS production and suppress inflammation, which may improve wound healing that is exacerbated by hyperglycemia.

## Figures and Tables

**Table 1:** Clinical studies of antioxidant application with non-surgical periodontal therapy.

Authors (Year)	Study Type	Treatment	Treatment Duration	Sample Type	Results
Abou Sulaiman and Shehadeh (2010) [77]	RCT	SRP with vitamin C (2000 mg/d)	1 month	30 patients with ChP 30 healthy controls	Significantly lower TAOC levels in patients with ChP SRP increased plasma TAOC and improve clinical measures in patients with ChP No additional effect of the adjunctive vitamin C on clinical parameters and TAOC levels when compared to SRP alone
Mathur et al. (2013) [79]	CH	SRP SRP with antioxidants (6 mg/d; lycopene, zinc, and selenium) Antioxidant only	3 doses in 2 weeks	30 patients with ChP 30 gingivitis patients 10 healthy controls	Low UA levels in gingivitis and patients with ChP (significantly lower in patients with more loss of periodontal attachments) No significant difference in UA levels between SRP alone and SRP with antioxidant. However, an increase in the mean UA levels was observed as elapsed time Antioxidant treatment significantly increased UA levels within 45 days in both gingivitis and patients with ChP
Raut and Sethi (2016) [81]	CH	SRP with coenzyme Q10 SRP with tea tree oil gel SRP with placebo gel	7 days	15 patients with ChP (moderate to severe)	Both antioxidant were effective in improving clinical markers of ChP (PI, GI, PPD, and CAL) Only the group treated with tea tree oil gel reported a slight change in taste
Kunsongkeit (2019) [78]	A double-blind, placebo-controlled, clinical trial	SRP with vitamin C (500 mg/day) SRP with placebo	2 months	31 patients with ChP	There was no significant difference between groups in terms of either FBS or HbA1c All periodontal parameters (PI, SBI, GI, PD, and CAL) were significantly improved from baseline in both groups. However, no significant difference was found between groups.
Taalab [80] (2021)	RCT	SRP alone SRP with locally delivered 5% tea tree oil gel	6 months	30 patients with ChP	A significant improvement of all clinical (PPD, CAL, GI, and BOP) and biochemical parameters (MMP-8) was observed in both groups A significant difference between the two groups was found in terms of both clinical and biochemical parameters.
Kaipa (2022) [82]	CH	SRP with Spirulina microspheres SRP with placebo	90 days	60 patients with ChP	The clinical parameters(GI, BOP, PPD, and CAL) were statistically significant in the experimental group compared to the control group. The salivary MDA levels were statistically significant in the experimental group compared to the control group. The serum MDA levels were reduced in both groups, but they were not statistically significant.

RCT, randomized controlled trial; ChP, chronic periodontitis; SRP, scaling and root planing; TAOC, total antioxidant capacity; CH, cohort study; UA, uric acid; PI, plaque index; GI, gingival index; PPD, probing pocket depth; CAL, clinical attachment level; BOP, bleeding on probing; SBI, Sulcus Bleeding Index; MMP-8, matrix metalloproteinase-8; MDA, malondialdehyde.

## Data Availability

Data sharing is not applicable to this article.

## References

[1] Lushchak V.I. (2014). Free radicals, reactive oxygen species, oxidative stress and its classification. Chem. Biol. Interact..

[2] Chapple I.L., Matthews J.B. (2007). The role of reactive oxygen and antioxidant species in periodontal tissue destruction. Periodontology 2000.

[3] Staudte H., Guntsch A., Volpel A., Sigusch B.W. (2010). Vitamin C attenuates the cytotoxic effects of Porphyromonas gingivalis on human gingival fibroblasts. Arch. Oral. Biol..

[4] Golz L., Memmert S., Rath-Deschner B., Jager A., Appel T., Baumgarten G., Gotz W., Frede S. (2014). LPS from *P. gingivalis* and hypoxia increases oxidative stress in periodontal ligament fibroblasts and contributes to periodontitis. Mediat. Inflamm..

[5] Jepsen S., Caton J.G., Albandar J.M., Bissada N.F., Bouchard P., Cortellini P., Demirel K., de Sanctis M., Ercoli C., Fan J. (2018). Periodontal manifestations of systemic diseases and developmental and acquired conditions: Consensus report of workgroup 3 of the 2017 World Workshop on the Classification of Periodontal and Peri-Implant Diseases and Conditions. J. Clin. Periodontol..

[6] Graziani F., Gennai S., Solini A., Petrini M. (2018). A systematic review and meta-analysis of epidemiologic observational evidence on the effect of periodontitis on diabetes An update of the EFP-AAP review. J. Clin. Periodontol..

[7] Pendyala G., Thomas B., Joshi S.R. (2013). Evaluation of Total Antioxidant Capacity of Saliva in Type 2 Diabetic Patients with and without Periodontal Disease: A Case-Control Study. N. Am. J. Med. Sci..

[8] Mizutani K., Park K., Mima A., Katagiri S., King G.L. (2014). Obesity-associated Gingival Vascular Inflammation and Insulin Resistance. J. Dent. Res..

[9] Schmidt A.M., Weidman E., Lalla E., Yan S.D., Hori O., Cao R., Brett J.G., Lamster I.B. (1996). Advanced glycation endproducts (AGEs) induce oxidant stress in the gingiva: A potential mechanism underlying accelerated periodontal disease associated with diabetes. J. Periodontal Res..

[10] Chopra A., Jayasinghe T.N., Eberhard J. (2022). Are Inflamed Periodontal Tissues Endogenous Source of Advanced Glycation End-Products (AGEs) in Individuals with and without Diabetes Mellitus? A Systematic Review. Biomolecules.

[11] Chen M., Cai W., Zhao S., Shi L., Chen Y., Li X., Sun X., Mao Y., He B., Hou Y. (2019). Oxidative stress-related biomarkers in saliva and gingival crevicular fluid associated with chronic periodontitis: A systematic review and meta-analysis. J. Clin. Periodontol..

[12] Allen E.M., Matthews J.B., O’ Halloran D.J., Griffiths H.R., Chapple I.L. (2011). Oxidative and inflammatory status in Type 2 diabetes patients with periodontitis. J. Clin. Periodontol..

[13] Ray P.D., Huang B.W., Tsuji Y. (2012). Reactive oxygen species (ROS) homeostasis and redox regulation in cellular signaling. Cell Signal.

[14] Almerich-Silla J.M., Montiel-Company J.M., Pastor S., Serrano F., Puig-Silla M., Dasi F. (2015). Oxidative Stress Parameters in Saliva and Its Association with Periodontal Disease and Types of Bacteria. Dis. Markers.

[15] Sczepanik F.S.C., Grossi M.L., Casati M., Goldberg M., Glogauer M., Fine N., Tenenbaum H.C. (2020). Periodontitis is an inflammatory disease of oxidative stress: We should treat it that way. Periodontology 2000.

[16] Landzberg M., Doering H., Aboodi G.M., Tenenbaum H.C., Glogauer M. (2015). Quantifying oral inflammatory load: Oral neutrophil counts in periodontal health and disease. J. Periodontal Res..

[17] Liu C., Mo L., Niu Y., Li X., Zhou X., Xu X. (2017). The Role of Reactive Oxygen Species and Autophagy in Periodontitis and Their Potential Linkage. Front. Physiol..

[18] Kanzaki H., Wada S., Narimiya T., Yamaguchi Y., Katsumata Y., Itohiya K., Fukaya S., Miyamoto Y., Nakamura Y. (2017). Pathways that Regulate ROS Scavenging Enzymes, and Their Role in Defense Against Tissue Destruction in Periodontitis. Front. Physiol..

[19] Miyasaki K.T. (1991). The neutrophil: Mechanisms of controlling periodontal bacteria. J. Periodontol..

[20] Wang Y., Andrukhov O., Rausch-Fan X. (2017). Oxidative Stress and Antioxidant System in Periodontitis. Front. Physiol..

[21] Banasova L., Kamodyova N., Jansakova K., Tothova L., Stanko P., Turna J., Celec P. (2015). Salivary DNA and markers of oxidative stress in patients with chronic periodontitis. Clin. Oral. Investig..

[22] Su H., Gornitsky M., Velly A.M., Yu H., Benarroch M., Schipper H.M. (2009). Salivary DNA, lipid, and protein oxidation in nonsmokers with periodontal disease. Free Radic. Biol. Med..

[23] Konopka T., Krol K., Kopec W., Gerber H. (2007). Total antioxidant status and 8-hydroxy-2′-deoxyguanosine levels in gingival and peripheral blood of periodontitis patients. Arch. Immunol. Et Ther. Exp..

[24] Nassar H., Kantarci A., van Dyke T.E. (2007). Diabetic periodontitis: A model for activated innate immunity and impaired resolution of inflammation. Periodontology 2000.

[25] Ohgi S., Johnson P.W. (1996). Glucose modulates growth of gingival fibroblasts and periodontal ligament cells: Correlation with expression of basic fibroblast growth factor. J. Periodontal Res..

[26] Willershausen-Zonnchen B., Lemmen C., Hamm G. (1991). Influence of high glucose concentrations on glycosaminoglycan and collagen synthesis in cultured human gingival fibroblasts. J. Clin. Periodontol..

[27] Buranasin P., Mizutani K., Iwasaki K., Pawaputanon Na Mahasarakham C., Kido D., Takeda K., Izumi Y. (2018). High glucose-induced oxidative stress impairs proliferation and migration of human gingival fibroblasts. PLoS ONE.

[28] Kido D., Mizutani K., Takeda K., Mikami R., Matsuura T., Iwasaki K., Izumi Y. (2017). Impact of diabetes on gingival wound healing via oxidative stress. PLoS ONE.

[29] Desta T., Li J., Chino T., Graves D.T. (2010). Altered fibroblast proliferation and apoptosis in diabetic gingival wounds. J. Dent. Res..

[30] Zhu C., Shen S., Zhang S., Huang M., Zhang L., Chen X. (2022). Autophagy in Bone Remodeling: A Regulator of Oxidative Stress. Front. Endocrinol..

[31] Agidigbi T.S., Kim C. (2019). Reactive Oxygen Species in Osteoclast Differentiation and Possible Pharmaceutical Targets of ROS-Mediated Osteoclast Diseases. Int. J. Mol. Sci..

[32] Liu R., Bal H.S., Desta T., Krothapalli N., Alyassi M., Luan Q., Graves D.T. (2006). Diabetes enhances periodontal bone loss through enhanced resorption and diminished bone formation. J. Dent. Res..

[33] Terheyden H., Lang N.P., Bierbaum S., Stadlinger B. (2012). Osseointegration--communication of cells. Clin. Oral. Implants Res..

[34] Saito N., Mikami R., Mizutani K., Takeda K., Kominato H., Kido D., Ikeda Y., Buranasin P., Nakagawa K., Takemura S. (2022). Impaired dental implant osseointegration in rat with streptozotocin-induced diabetes. J. Periodontal Res..

[35] Mouraret S., Hunter D.J., Bardet C., Brunski J.B., Bouchard P., Helms J.A. (2014). A pre-clinical murine model of oral implant osseointegration. Bone.

[36] Du Z., Lee R.S., Hamlet S., Doan N., Ivanovski S., Xiao Y. (2016). Evaluation of the first maxillary molar post-extraction socket as a model for dental implant osseointegration research. Clin. Oral. Implants Res..

[37] Yu S.H., Hao J., Fretwurst T., Liu M., Kostenuik P., Giannobile W.V., Jin Q. (2018). Sclerostin-Neutralizing Antibody Enhances Bone Regeneration Around Oral Implants. Tissue Eng. Part. A.

[38] Hou M., Lee R.S.B., Du Z., Hamlet S.M., Vaquette C., Ivanovski S. (2019). The influence of high-dose systemic zoledronate administration on osseointegration of implants with different surface topography. J. Periodontal Res..

[39] Feng Y.F., Wang L., Zhang Y., Li X., Ma Z.S., Zou J.W., Lei W., Zhang Z.Y. (2013). Effect of reactive oxygen species overproduction on osteogenesis of porous titanium implant in the present of diabetes mellitus. Biomaterials.

[40] Sies H. (1997). Oxidative stress: Oxidants and antioxidants. Exp. Physiol..

[41] Ellis S.D., Tucci M.A., Serio F.G., Johnson R.B. (1998). Factors for progression of periodontal diseases. J. Oral. Pathol. Med..

[42] Xi X., Li Z.X., Zhao Y., Liu H., Chen S., Liu D.X. (2022). N-acetylcysteine promotes cyclic mechanical stress-induced osteogenic differentiation of periodontal ligament stem cells by down-regulating Nrf2 expression. J. Dent. Sci..

[43] Pei Y., Liu H., Yang Y., Yang Y., Jiao Y., Tay F.R., Chen J. (2018). Biological Activities and Potential Oral Applications of N-Acetylcysteine: Progress and Prospects. Oxid. Med. Cell Longev..

[44] Ueno T., Yamada M., Igarashi Y., Ogawa T. (2011). N-acetyl cysteine protects osteoblastic function from oxidative stress. J. Biomed. Mater. Res. A.

[45] Orihuela-Campos R.C., Tamaki N., Mukai R., Fukui M., Miki K., Terao J., Ito H.O. (2015). Biological impacts of resveratrol, quercetin, and N-acetylcysteine on oxidative stress in human gingival fibroblasts. J. Clin. Biochem. Nutr..

[46] Kim D.Y., Jun J.H., Lee H.L., Woo K.M., Ryoo H.M., Kim G.S., Baek J.H., Han S.B. (2007). N-acetylcysteine prevents LPS-induced pro-inflammatory cytokines and MMP2 production in gingival fibroblasts. Arch. Pharm. Res..

[47] Kominato H., Takeda K., Mizutani K., Mikami R., Kido D., Buranasin P., Saito N., Takemura S., Nakagawa K., Nagasawa T. (2022). Metformin accelerates wound healing by Akt phosphorylation of gingival fibroblasts in insulin-resistant prediabetes mice. J. Periodontol..

[48] Mikami R., Mizutani K., Shioyama H., Matsuura T., Aoyama N., Suda T., Kusunoki Y., Takeda K., Izumi Y., Aida J. (2022). Influence of aging on periodontal regenerative therapy using enamel matrix derivative: A 3-year prospective cohort study. J. Clin. Periodontol..

[49] Miron R.J., Sculean A., Cochran D.L., Froum S., Zucchelli G., Nemcovsky C., Donos N., Lyngstadaas S.P., Deschner J., Dard M. (2016). Twenty years of enamel matrix derivative: The past, the present and the future. J. Clin. Periodontol..

[50] Hammarström L. (1997). Enamel matrix, cementum development and regeneration. J. Clin. Periodontol..

[51] Nokhbehsaim M., Deschner B., Winter J., Bourauel C., Jager A., Jepsen S., Deschner J. (2012). Anti-inflammatory effects of EMD in the presence of biomechanical loading and interleukin-1beta in vitro. Clin. Oral. Investig..

[52] Villa O., Wohlfahrt J.C., Koldsland O.C., Brookes S.J., Lyngstadaas S.P., Aass A.M., Reseland J.E. (2016). EMD in periodontal regenerative surgery modulates cytokine profiles: A randomised controlled clinical trial. Sci. Rep..

[53] Gestrelius S., Andersson C., Lidstrom D., Hammarstrom L., Somerman M. (1997). In vitro studies on periodontal ligament cells and enamel matrix derivative. J. Clin. Periodontol..

[54] Keila S., Nemcovsky C.E., Moses O., Artzi Z., Weinreb M. (2004). In vitro effects of enamel matrix proteins on rat bone marrow cells and gingival fibroblasts. J. Dent. Res..

[55] Takeda K., Mizutani K., Matsuura T., Kido D., Mikami R., Noda M., Buranasin P., Sasaki Y., Izumi Y. (2018). Periodontal regenerative effect of enamel matrix derivative in diabetes. PLoS ONE.

[56] Takeda K., Mizutani K., Matsuura T., Kido D., Mikami R., Buranasin P., Saito N., Kominato H., Takemura S., Nakagawa K. (2022). Antioxidant effect of enamel matrix derivative for early phase of periodontal tissue regeneration in diabetes. J. Periodontol..

[57] Fijany A., Sayadi L.R., Khoshab N., Banyard D.A., Shaterian A., Alexander M., Lakey J.R.T., Paydar K.Z., Evans G.R.D., Widgerow A.D. (2019). Mesenchymal stem cell dysfunction in diabetes. Mol. Biol. Rep..

[58] Luo M.L., Jiao Y., Gong W.P., Li Y., Niu L.N., Tay F.R., Chen J.H. (2020). Macrophages enhance mesenchymal stem cell osteogenesis via down-regulation of reactive oxygen species. J. Dent..

[59] Yamawaki I., Taguchi Y., Komasa S., Tanaka A., Umeda M. (2017). Effects of glucose concentration on osteogenic differentiation of type II diabetes mellitus rat bone marrow-derived mesenchymal stromal cells on a nano-scale modified titanium. J. Periodontal Res..

[60] Mizutani K., Shioyama H., Matsuura T., Mikami R., Takeda K., Izumi Y., Aoki A., Iwata T. (2021). Periodontal regenerative therapy in patients with type 2 diabetes using minimally invasive surgical technique with enamel matrix derivative under 3-year observation: A prospective cohort study. J. Periodontol..

[61] Gharbi A., Hamila A., Bouguezzi A., Dandana A., Ferchichi S., Chandad F., Guezguez L., Miled A. (2019). Biochemical parameters and oxidative stress markers in Tunisian patients with periodontal disease. BMC Oral. Health.

[62] Paredes-Sanchez E., Montiel-Company J.M., Iranzo-Cortes J.E., Almerich-Torres T., Bellot-Arcis C., Almerich-Silla J.M. (2018). Meta-Analysis of the Use of 8-OHdG in Saliva as a Marker of Periodontal Disease. Dis. Markers.

[63] Baltacioglu E., Sukuroglu E. (2019). Protein carbonyl levels in serum, saliva and gingival crevicular fluid in patients with chronic and aggressive periodontitis. Saudi Dent. J..

[64] Panjamurthy K., Manoharan S., Ramachandran C.R. (2005). Lipid peroxidation and antioxidant status in patients with periodontitis. Cell Mol. Biol. Lett..

[65] Toczewska J., Baczyńska D., Zalewska A., Maciejczyk M., Konopka T. (2023). The mRNA expression of genes encoding selected antioxidant enzymes and thioredoxin, and the concentrations of their protein products in gingival crevicular fluid and saliva during periodontitis. Dent. Med. Probl..

[66] Baumgartner-Parzer S.M., Wagner L., Pettermann M., Grillari J., Gessl A., Waldhausl W. (1995). High-glucose--triggered apoptosis in cultured endothelial cells. Diabetes.

[67] Giugliano D., Ceriello A., Paolisso G. (1996). Oxidative stress and diabetic vascular complications. Diabetes Care.

[68] Paolisso G., Giugliano D. (1996). Oxidative stress and insulin action: Is there a relationship?. Diabetologia.

[69] Sculley D.V., Langley-Evans S.C. (2003). Periodontal disease is associated with lower antioxidant capacity in whole saliva and evidence of increased protein oxidation. Clin. Sci..

[70] Simon H.U., Haj-Yehia A., Levi-Schaffer F. (2000). Role of reactive oxygen species (ROS) in apoptosis induction. Apoptosis.

[71] Evans J.L., Goldfine I.D., Maddux B.A., Grodsky G.M. (2003). Are oxidative stress-activated signaling pathways mediators of insulin resistance and beta-cell dysfunction?. Diabetes.

[72] Sanz M., Ceriello A., Buysschaert M., Chapple I., Demmer R.T., Graziani F., Herrera D., Jepsen S., Lione L., Madianos P. (2018). Scientific evidence on the links between periodontal diseases and diabetes: Consensus report and guidelines of the joint workshop on periodontal diseases and diabetes by the International Diabetes Federation and the European Federation of Periodontology. J. Clin. Periodontol..

[73] Altıngöz S.M., Kurgan Ş., Önder C., Serdar M.A., Ünlütürk U., Uyanık M., Başkal N., Tatakis D.N., Günhan M. (2021). Salivary and serum oxidative stress biomarkers and advanced glycation end products in periodontitis patients with or without diabetes: A cross-sectional study. J. Periodontol..

[74] Bullon P., Newman H.N., Battino M. (2014). Obesity, diabetes mellitus, atherosclerosis and chronic periodontitis: A shared pathology via oxidative stress and mitochondrial dysfunction?. Periodontology 2000.

[75] Smiley C.J., Tracy S.L., Abt E., Michalowicz B.S., John M.T., Gunsolley J., Cobb C.M., Rossmann J., Harrel S.K., Forrest J.L. (2015). Systematic review and meta-analysis on the nonsurgical treatment of chronic periodontitis by means of scaling and root planing with or without adjuncts. J. Am. Dent. Assoc..

[76] Mailoa J., Lin G.H., Khoshkam V., MacEachern M., Chan H.L., Wang H.L. (2015). Long-Term Effect of Four Surgical Periodontal Therapies and One Non-Surgical Therapy: A Systematic Review and Meta-Analysis. J. Periodontol..

[77] Abou Sulaiman A.E., Shehadeh R.M. (2010). Assessment of total antioxidant capacity and the use of vitamin C in the treatment of non-smokers with chronic periodontitis. J. Periodontol..

[78] Kunsongkeit P., Okuma N., Rassameemasmaung S., Chaivanit P. (2019). Effect of Vitamin C as an Adjunct in Nonsurgical Periodontal Therapy in Uncontrolled Type 2 Diabetes Mellitus Patients. Eur. J. Dent..

[79] Mathur A., Mathur L., Manohar B., Mathur H., Shankarapillai R., Shetty N., Bhatia A. (2013). Antioxidant therapy as monotherapy or as an adjunct to treatment of periodontal diseases. J. Indian. Soc. Periodontol..

[80] Taalab M.R., Mahmoud S.A., Moslemany R.M.E., Abdelaziz D.M. (2021). Intrapocket application of tea tree oil gel in the treatment of stage 2 periodontitis. BMC Oral. Health.

[81] Raut C.P., Sethi K.S. (2016). Comparative evaluation of co-enzyme Q10 and Melaleuca alternifolia as antioxidant gels in treatment of chronic periodontitis: A clinical study. Contemp. Clin. Dent..

[82] Kaipa V.R.K., Asif S.M., Assiri K.I., Saquib S.A., Arem S.A., Sree S., Yassin S.M., Ibrahim M., Shariff M., Shamsudeen S.M. (2022). Antioxidant effect of spirulina in chronic periodontitis. Medicine.

[83] Merle C.L., Lenzen C., Schmalz G., Ziebolz D. (2023). Systematic Review on Protocols of Coenzyme Q10 Supplementation in Non-Surgical Periodontitis Therapy. Nutrients.

[84] Chatzopoulos G.S., Karakostas P., Kavakloglou S., Assimopoulou A., Barmpalexis P., Tsalikis L. (2022). Clinical Effectiveness of Herbal Oral Care Products in Periodontitis Patients: A Systematic Review. Int. J. Environ. Res. Public Health.

[85] Borgnakke W.S., Ylostalo P.V., Taylor G.W., Genco R.J. (2013). Effect of periodontal disease on diabetes: Systematic review of epidemiologic observational evidence. J. Clin. Periodontol..

[86] Mizutani K., Buranasin P., Mikami R., Takeda K., Kido D., Watanabe K., Takemura S., Nakagawa K., Kominato H., Saito N. (2021). Effects of Antioxidant in Adjunct with Periodontal Therapy in Patients with Type 2 Diabetes: A Systematic Review and Meta-Analysis. Antioxidants.

[87] Mizutani K., Mikami R., Tsukui A., Nagai S., Pavlic V., Komada W., Iwata T., Aoki A. (2022). Novel flapless esthetic procedure for the elimination of extended gingival metal tattoos adjacent to prosthetic teeth: Er:YAG laser micro-keyhole surgery. J. Prosthodont. Res..

[88] Mijiritsky E., Ferroni L., Gardin C., Peleg O., Gultekin A., Saglanmak A., Delogu L.G., Mitrecic D., Piattelli A., Tatullo M. (2019). Presence of ROS in Inflammatory Environment of Peri-Implantitis Tissue: In Vitro and In Vivo Human Evidence. J. Clin. Med..

[89] González-Serrano J., López-Pintor R.M., Serrano J., Torres J., Hernández G., Sanz M. (2021). Short-term efficacy of a gel containing propolis extract, nanovitamin C and nanovitamin E on peri-implant mucositis: A double-blind, randomized, clinical trial. J. Periodontal Res..

[90] Li X., Tang L., Lin Y.F., Xie G.F. (2018). Role of vitamin C in wound healing after dental implant surgery in patients treated with bone grafts and patients with chronic periodontitis. Clin. Implant. Dent. Relat. Res..

[91] Dreyer H., Grischke J., Tiede C., Eberhard J., Schweitzer A., Toikkanen S.E., Glöckner S., Krause G., Stiesch M. (2018). Epidemiology and risk factors of peri-implantitis: A systematic review. J. Periodontal Res..

[92] Elangovan S., Brogden K.A., Dawson D.V., Blanchette D., Pagan-Rivera K., Stanford C.M., Johnson G.K., Recker E., Bowers R., Haynes W.G. (2014). Body fat indices and biomarkers of inflammation: A cross-sectional study with implications for obesity and peri-implant oral health. Int. J. Oral. Maxillofac. Implant..

